# The Nordic Nutrition Recommendations 2022 – food consumption and nutrient intake in the adult population of the Nordic and Baltic countries

**DOI:** 10.29219/fnr.v66.8572

**Published:** 2022-06-08

**Authors:** Eva Warensjö Lemming, Tagli Pitsi

**Affiliations:** 1 Department of Risk and Benefit Assessment, Swedish Food Agency, Uppsala, Sweden; 2 Departments of Food Studies, Nutrition and Dietetics and Surgical Sciences, Medical Epidemiology, Uppsala University, Uppsala, Sweden; 3 Nutrition and Exercise Unit, Centre for Health Risk Prevention, National Institute for Health Development, Tallinn, Estonia; 4 Department of Chemistry and Biotechnology, Tallinn University of Technology, Talinn, Estonia; 5 Haapsalu College, Tallinn University, Haapsalu, Estonia

**Keywords:** Nordic and Baltic countries, Nordic nutrition recommendations, food consumption, nutrient intake, national dietary survey

## Abstract

**Background:**

Knowledge about the nutrient intakes and food consumption in the Nordic and Baltic countries is important for the formulation of dietary reference values (DRVs) and food-based dietary guidelines (FBDGs), as part of the Nordic Nutrition Recommendations 2022 project (NNR2022).

**Objective:**

To describe nutrient intake and food consumption at a broad level in the adult population of each Nordic and Baltic country. This paper also provides guidance on where to find more information on the nutrient intake and food consumption reported from each country.

**Design:**

Information about the dietary surveys as well as the daily mean intakes was retrieved from the national dietary surveys in each of the Nordic and Baltic countries. Tabulation of the population intakes divided by sex for macronutrients, 20 micronutrients, and for the following broader food groups, Beverages, Cereals, Potatoes, Vegetables, Fruits and berries, Fish and seafood, Meat and meat products, Milk and dairy products, Cheese, Eggs, Fats and oils, and Sweets and sweet bakery products, was done.

**Results and Discussion:**

The Nordic and Baltic countries share not only similarities but also differences in food consumption patterns, which is reflected in differences in average food consumption and nutrient intakes between the countries. This may be related to the dietary assessment method, prevalence of misreporting, and participation rates in the different dietary surveys. Other factors that may play a role are differences in the calculation procedures in the food composition databases and the definition of food groups.

**Conclusion:**

The nutrient intake and, especially, food consumption differ between the Nordic and Baltic countries because of differences in food patterns and factors related to the dietary surveying, food grouping, and calculation procedures in each country. To facilitate future comparisons between countries, it would be of interest to harmonize food groupings and the age groups reported on.

## Popular scientific summary

- Food consumption and nutrient intakes are collected in national dietary surveys in each of the Nordic and Baltic countries.
- This article presents food and nutrient intake data in the adult population of each Nordic and Baltic country.
- For the formulation of dietary reference values and food-based dietary guidelines within the Nordic Nutrition Recommendations 2022 project, these data will be considered.

The 5th edition of the Nordic Nutrition Recommendations (NNR 2012) that was published in 2014 is currently being revised (1). The NNR constitutes the scientific basis for national dietary reference values (DRVs) overall, and partly for the food-based dietary guidelines (FBDGs) in Denmark, Finland, Iceland, Norway, and Sweden, as well as in Estonia, Latvia, and Lithuania. The DRVs are intended for the general population, considering different age groups and pregnant and lactating women. The framework for the development of the 6th edition (NNR 2022) of the NNR (1) has been described in four articles so far (2–5).

Dietary habits and nutrient intakes are assessed in dietary surveys all across the world. At the national level, dietary surveys have been carried out for decades in the Nordic countries, and the surveying in the Baltic countries has also been going on since the 1990s. A comprehensive review of national dietary surveys in the European region has been published previously (6). The information on food consumption collected in the dietary survey is converted to nutrient intakes by calculation procedures and linkage to food composition databases. The surveys generate data on average daily food consumption and nutrient intakes. The distribution of intakes and the proportion of consumers of selected food items can also be calculated. These estimates are used to evaluate the adequacy of nutrient intakes according to DRVs and adherence to FBDGs. The results from a national dietary survey can also reveal common nutritional problems as well as potential diet-related health problems in a population (7).

This article aims to describe and compare nutrient intake and food consumption in the Nordic and Baltic countries at a broad level in the adult population. This article also provides guidance on where to find more specific information on food consumption and nutrient intakes in the Nordic and Baltic countries and serves as a background document for the formulation of DRVs and FBDGs within the NNR 2022 project.

## Materials and methods

Data on the characteristics of study participants, survey information, dietary assessment method, and intake of energy and nutrients, as well as the consumption of foods were retrieved from the latest representative national dietary surveys conducted among adults in the eight countries (8–17). For Latvia and Lithuania, information had to be retrieved from different surveys since all information on food and nutrients was not available in the latest survey. Nutrients with a DRV in NNR 2012 were considered and were the following: Protein, Fats, Saturated fatty acids (SFA), Monounsaturated fatty acids (MUFA), Polyunsaturated fatty acids (PUFA), Carbohydrates (Ch), Fiber, Alcohol, Protein, Cholesterol, Vitamin A, Vitamin D, Vitamin E, Vitamin B1, Vitamin B2, Niacin, Vitamin B6, Folate, Vitamin B12, Vitamin C, Sodium, Potassium, Calcium, Phosphorus, Magnesium, Iron, Zinc, Copper, Selenium, and Iodine. In some countries, information of certain nutrient intakes was lacking and, therefore, will be missing in the tables.

Mean intakes (excluding food supplements) per day of nutrients were collected from the surveys, while standard deviations (SD) were not available from all surveys. The daily consumption of the broader food groups (in bold) and the specific food groups is as follows: **Beverages**, Soft drinks, Cordials, etc., Juice, Coffee, Tea, **Cereals**, Bread, Other grains, Breakfast cereals, Porridge, **Potatoes**, **Vegetables**, Pulses (legumes), **Fruits and berries**, **Nuts and seeds**, **Fish and seafood**, **Meat and meat products**, Red meat, Poultry, **Milk and dairy products**, **Cheese**, **Eggs**, **Fats and oils**, Spreads, Margarine, Vegetable oil, **Sweets and sweet bakery products**, Cakes and biscuits, and Sweets. The mean and SD (if available) or 95% confidence intervals (CIs) (in Finland) are reported. The food group definitions differ between the countries. This means that foods behind the food groups may differ, and the level of information may differ. For example, Finland has information only for Cereals, while Sweden has also for Cereal subgroups, such as bread, breakfast cereals, and porridge. In those cases where we were not able to retrieve information on food group consumption or nutrient intake from the survey reports, we were in contact with the dietary survey teams that had collected the data. In some instances, SDs and 95% CIs were not possible to retrieve, and then only the mean values are reported. The aim was to compare nutrient intakes and food consumption between the countries. Furthermore, daily mean intakes of micronutrients and macronutrients were compared with the DRVs, recommended intake (RI), and RI ranges, in the NNR 2012 (1). However, it should be kept in mind that comparing mean micronutrient intakes with the RIs is not the method recommended when assessing nutrient intakes of a population. A mean intake above the RI of a given micronutrient indicates a probable low prevalence of inadequacy in the population group; however, this does not exclude inadequate intakes in some individuals. Moreover, if the mean intake is below the RI, it is not possible to draw any strong conclusion regarding the prevalence of inadequacy at the group level. If a proper risk assessment of the nutrient intake in a population group should be done, the important DRV to consider is the average requirement (AR). Data on the usual nutrient intakes reported as percentiles are then required. More information on how to do a proper assessment of the adequacy of the intakes of micronutrients on the population level and the use of DRVs is presented in Trolle et al. (Unpublished observation). A full risk assessment of the micronutrient intakes in the Nordic and Baltic countries is beyond the scope of this article.

## Results

Information about the national dietary surveys among adults in the Nordic and Baltic countries is given in Table 1. The surveys cover the adult population in the Nordic and Baltic countries. The smallest age range for the adult population was between 17 and 64 years in Latvia, and the highest age range was between 18 and 80 years in Sweden and Iceland. The dietary surveys in Finland, Estonia, and Latvia were undertaken as part of the EU-Menu project (18), partly funded by the European Food Safety Authority (EFSA). Latvian data are based on two surveys. Access to all respective survey results can be done via the URL provided in Table 1. For the Estonian data, no main report with survey results was published. The survey results can, however, be retrieved from an online database by the URL provided in Table 1. The results from the survey conducted in Lithuania in 2019 were not published as a report previously and were received directly from the Lithuanian dietary survey team (Personal communication, Gabija Bulotaitė). Participation rate differed between 27% in Estonia (15) and 90% in Latvia. Most countries had used the 2 × 24 h recall method to collect the data, including 2 non-consecutive days, while Sweden used a web-based food record covering 4 days and Denmark, a 7-day food record. The surveys were carried out between 2007 and 2020.

**Table 1:** The latest national dietary surveys among adults in the Nordic and Baltic countries

Country	Survey	Dietary assessment method	Year	Age groups	Number of participants	Participation rate (%)	Ref.
Denmark	Danskernes kostvaner 2011–2013^1^	7-d food record	2011–13	18–75	3,016	52	(10)
Finland	FINDIET, FinRavinto 2017^2^	2*24 h recall	2017	25–74	3,099	53	(14, 17)
Iceland	Hvað borða ĺslendingar?^3^	2*24 h recall	2010–11	18–80	1,312	69	(16)
Norway	Norkost 3^4^	2*24 h recall	2010–11	18–70	1,787	37	(11)
Sweden	Riksmaten vuxna 2010–11^5^	4-d food record, web based	2010–11	18–80	1,797	36	(8)
Estonia	Eesti rahvastiku toitumise uuring 2014, Estonian National Dietary Survey 2014^6^	2*24 h recall	2014	18–74	2,713	~33	(15)
Latvia	Latvian National Dietary Survey on the general population^7^	2*24 h recall	2007–09	17–64	1,377	~90	(12, 13)
	Study of salt and iodine consumption in the adult population in Latvia^8^	2*24 h recall	2018–2020	19–64	1,011	54	
Lithuania	Study of actual nutrition and nutrition habits of Lithuanian adult population^9^	2*24 h recall	2013–2014	19–75	2,513	61	(9, 24)
	Study of actual nutrition, dietary, and physical activity habits and knowledge of nutrition and physical activity of Lithuanian adult and elderly population^10^	2*24 h recall	2019	19–75	2,910		

1 http://www.food.dtu.dk/-/media/Institutter/Foedevareinstituttet/Publikationer/Pub 2015/Rapport_Danskernes-Kostvaner-2011-2013.ashx?la=da.

2 http://urn.fi/URN:ISBN:978-952-343-238-3.

3 https://www.landlaeknir.is/servlet/file/store93/item14901/Hva%C3%B0%20bor%C3%B0a%20%C3%8Dslendingar_april%202012.pdf.

4 https://www.helsedirektoratet.no/rapporter/norkost-3-en-landsomfattende-kostholdsundersokelse-blant-menn-og-kvinner-i-norge-i-alderen-18-70-ar-2010-11/Norkost%203%20en%20landsomfattende%20kostholdsundersokelse%20blant%20menn%20og%20kvinner%20i%20Norge%20i%20alderen-18-70%20%C3%A5r%202010-11.pdf/_/attachment/inline/b7bafaab-6059-4450-8d76-c3ed9f3eaf3f:be251cd1153cf1ae8e4c46eedddc13b36da3d11d/Norkost%203%20en%20landsomfattende%20kostholdsundersokelse%20blant%20menn%20og%20kvinner%20i%20Norge%20i%20alderen-18-70%20%C3%A5r%202010-11.pdf.

5 https://www.livsmedelsverket.se/globalassets/publikationsdatabas/rapporter/2011/riksmaten_2010_20111.pdf?AspxAutoDetectCookieSupport=1.

6 https://efsa.onlinelibrary.wiley.com/doi/abs/10.2903/sp.efsa.2017.EN-1198

7 https://efsa.onlinelibrary.wiley.com/doi/epdf/10.2903/sp.efsa.2017.EN-1307 (Methods report Efsa).

8 https://esparveselibu.lv/petijums/sals-un-joda-paterins-latvijas-pieauguso-iedzivotaju-populacija.

9 https://hi.lt/uploads/pdf/visuomenes%20sveikata/2016.01.72/VS%202016%201(72)%20ORIG%20Mitybos%20iprociai.pdf.

10 Not published.

Mean intakes of macronutrients and micronutrients are presented in Tables 2–5. The percentage contribution from macronutrients to total energy intake in the adult populations of the Nordic and Baltic countries is shown in Tables 2 and 3. Reported energy intake ranged between 8.7 mega joule (MJ) (Estonia) and 11.2 MJ (Denmark) in men, and between 6.5 MJ (Estonia) and 8.4 MJ (Denmark) in women. The percentage contribution of macronutrients to total energy was roughly similar among the populations in the Nordic countries as well as in Estonia, with the carbohydrate contribution ranging between 43 and 48 energy percent (E%), the fat contribution ranging between 35 and 39 E%, and the protein contribution ranging between 15 and 19 E%. In all Nordic countries and Estonia, alcohol was included in the reported energy intake, with the highest reported intake of alcohol in Denmark. In Latvia and Lithuania, the contribution from alcohol was not included, and the reported energy contribution from fat was higher and lower from carbohydrates compared to the other countries. The percentage contribution from SFA was too high compared to the recommendation in all countries (Tables 2 and 3), with the highest intakes in Danish and Finnish men (15 E%) and the lowest intakes in Sweden and Norway (13 E%). Intake of PUFA was the highest in Finland (about 7 E%), and fiber intake was lower than the recommendation in all countries. Icelandic and Lithuanian women had the lowest intakes of fiber, 16 g per day.

**Table 2:** Daily mean intakes of energy, macronutrients, fatty acids, fiber, and cholesterol among adults in the Nordic countries^1^

	Denmark 2011 (18–75 years)		Finland 2017 (18–74 years)		Iceland 2010 (18–80 years)		Norway 2010 (18–70 years)		Sweden 2010 (18–80 years)	
	Men (*n* = 1,464)	Women (*n* = 1,552)	Men (*n* = 780)	Women (*n* = 875)	Men (*n* = 632)	Women (*n* = 680)	Men (*n* = 862)	Women (*n* = 925)	Men (*n* = 792)	Women (*n* = 1,005)
Energy, MJ	11.2	8.4	9.5	7.3	9.9	7.4	10.9	8	9.4	7.4
Protein, g	101	76	98	73	106	75	112	81	91.8	71.7
Fats, g	111	83	97	75	99	72	102	75	86.9	69.7
SFA, g	45	33	38	28	39.8	29.0	39	29	33.4	26.7
MUFA, g	41	31	37	28	31.8	23.0	34	25	32.6	26.0
PUFA, g	17	13	17	14	15.8	12	18	13	14	11.5
Carbohydrates, g	269	211	219	177	239	188	278	205	237	192
Fiber, g	24	21	22	20	17.7	16.0	26	22	21.3	18.8
Alcohol, g	20.1	11.2	7.9	2.9	8.7	4.4	10	6.3	13	7.3
Protein, E%	16	15	18.0	17.5	18.6	17.6	18	18	17	16.8
Fats, E%	36	36	38.7	37.7	36.7	35.9	34	34	34.0	34.4
Chs, all, E%	42.6	45.4	43.4	45.7	42.5	45.0	45.4	46.1	45.2	46.1
Alcohol, E%	5.3	3.9	2.4	1.1	2.3	1.5	2.5	2.1	3.9	2.8
Chs, available, E%	41	43	41.3	42.5	41.0	43.2	43.4	43.8	43.4	44
Fiber, E%	1.6	2.4	2.1	3.2	1.5	1.8	2	2.3	1.8	2.1
SFA, E%	15	14	15.1	14.4	14.6	14.3	13	13	13.0	13.1
MUFA, E%	13	13	14.6	14.3	11.8	11.4	12	12	12.8	12.9
PUFA, E%	5.5	5.6	6.8	6.9	5.9	6.0	6.3	6.2	5.5	5.7
Cholesterol, mg	–	–	289	231	392	269	398	301	320	263

1 Results given as reported by respective country.

**Table 3:** Daily mean intakes of energy, macronutrients, fatty acids, fiber, and cholesterol among adults in the Baltic countries^1^

	Estonia 2014 (18–74 years)		Latvia 2018 (19–64 years)		Lithuania 2019 (19–75 years)	
	Men (*n* = 907)	Women (*n* = 1,806)	Men (*n* = 470)	Women (*n* = 541)	Men (*n* = 1,348)	Women (*n* = 1,562)
Energy, MJ	8.7	6.5	10.0	7.4	8.7^2^	6.7^2^
Protein, g	86.5	63.2	104.4	75.1	78.7	62.8
Fats, g	83.5	63.3	108.3	80.2	103.1	76.4
SFA, g	32.5	25.1	38.1^3^	28.1^3^	33.0	24.5
MUFA, g	31.4	22.9	33.4^3^	24.0^3^	40.2	29.8
PUFA, g	14.4	10.7	14.8^3^	10.8^3^	23.7	17.6
Carbohydrates, g	236	190	231	176	210	168
Fiber, g	19.1	17.2	20.1	18	18.0	16
Alcohol, g	-	-	13.2	2.9	-	-
Protein, E%	16.6	16.2	17.4^2^	17.0^2^	15.6^2^	16.0^2^
Fats, E%	34.9	34.7	40.6^2^	40.8^2^	43.7^2^	42.1^2^
CHs, all, E%	44.9	48.1	38.5^2^	38.5^2^	40.4^2^	41.8^2^
Alcohol, E%	3.2	0.7	-	-	-	-
CHs, available, E%	-	-	-	-	-	-
Fiber, g	-	-	-	-	-	-
SFA, E%	13.5	13.7	-	-	14.0^2^	13.5^2^
MUFA, E%	13.1	12.6	-	-	-	-
PUFA, E%	6.04	5.94	-	-	-	-
Cholesterol, mg	338	247	-	-	330	274

1 Results given as reported by respective country, except for Estonia and Latvia (calculated from different age groups’ results on the population level, taking into account the numbers of participants in each age group).

2 Total energy does not include the energy from alcohol.

3 From National Survey, all participants’ average 7–64 years (*n* for men 1,001 and *n* for women 948).

Note: Energy intake was calculated from kcal to MJ by the factor 4.184 only for Latvia.

**Table 4:** Daily mean intakes of vitamins and minerals among adults as reported in the Nordic countries

	Denmark 2011 (18–75 years)		Finland 2017 (18–74 years)		Iceland 2010 (18–80 years)		Norway 2010 (18–70 years)		Sweden 2010 (18–80 years)	
	Men (*n* = 1,464)	Women (*n* = 1,552)	Men (*n* = 780)	Women (*n* = 875)	Men (*n* = 632)	Women (*n* = 680)	Men (*n* = 862)	Women (*n* = 925)	Men (*n* = 792)	Women (*n* = 1,005)
Vitamin A, retinol equivalents (RE)	1,556	1,110	911	747	1,346	960	1,011	769	812	829
Vitamin D, μg	5.3	4.3	13	10	9.7	6.6	6.7	4.9	7.6	6.4
Vitamin E, mg	9.5	8.8	11.8	10.2	11.6	9.4	12	10	13.2	11.7
Vitamin B_1_, mg	1.6	1.2	1.4	1.1	1.4	1.1	1.9	1.4	1.4	1.1
Vitamin B_2_, mg	2.0	1.6	2.0	1.6	2.0	1.4	2.1	1.6	1.7	1.4
Niacin, niacin equivalents (NE)	40	30	39	29	41	29	-	-	41	32
Vitamin B_6_, mg	1.8	1.4	2.2	1.8	1.8	1.4	1.9	1.5	2.3	1.8
Folate, µg	370	329	247	222	304	248	279	231	266	253
Vitamin B_12_, µg	8.0	5.6	6.6	4.9	8.4	5.5	8.9	6.0	6	5
Vitamin C, mg	113	115	98	111	103	101	105	111	93	96
Sodium, mg	4,400	3,200	3,334	2,491	3,772	2,600	3,600	2,500	3,591	2,746
Potassium, g	3.9	3.2	4	3.4	3.4	2.6	4.2	3.4	3.4	2.9
Calcium, mg	1,188	1,038	1,182	984	1,034	820	1,038	811	945	820
Phosphorus, mg	1,782	1,384	1,710	1,357	1,788	1,316	-	-	1,541	1,242
Magnesium, mg	424	342	404	337	335	263	439	346	364	305
Iron, mg	13	10	11	10	12.5	9.4	13.0	9.9	11.5	9.5
Zinc, mg	14.1	10.5	13	10	12.4	8.8	-	-	12.4	9.5
Copper, mg	-	-	1.3	1.1	1.3	1.1	-	-	-	-
Selenium, μg	61	46	88	68	83	60	-	-	50	42
Iodine, µg	268	227	229	185	195	142	-	-	-	-

Note: Color coding of cells: Green – above RI; yellow – below RI; red – high intake compared to the Nordic nutrition recommendations 2012 (1).

**Table 5:** Daily mean intakes of vitamins and minerals among adults as reported in the Baltic countries

	Estonia 2014 (18–74 years)		Latvia 2018 (17–64 years)		Lithuania 2019 (19–75 years)	
	Men (*n* = 907)	Women (*n* = 1,806)	Men (*n* = 470)	Women (*n* = 541)	Men (*n* = 1,348)	Women (*n* = 1,562)
Vitamin A, retinol equivalents (RE)	1,155	942	666^1^		1,053	934
Vitamin D, μg	5.7	4.3	7.2	9.1	5.5	4.8
Vitamin E, mg	9.4	7.8	10.8	12.2	14.9	12.9
Vitamin B_1_, mg	1.1	0.8	1.3^1^		1.4	1.0
Vitamin B_2_, mg	1.2	1.0	1.3^1^		1.4	1.2
Niacin, niacin equivalents (NE)	32.9	23.7	13.1^1^		16.3	12.7
Vitamin B_6_, mg	1.5	1.2	1.7^1^		1.9	1.6
Folates, µg	198	164	243	216	383	200
Vitamin B_12_, µg	8.0	5.8	3.7^1^		3.3	2.9
Vitamin C, mg	72	75	116	132	73	69
Sodium, mg	2,563	1,791	3,332	2,501	3,175	2,419
Potassium, g	3.8	3.0	3	2.5	2.9	2.4
Calcium, mg	768	633	767	659	660	546
Phosphorus, mg	1,392	1,061	1,186^2^	867^2^	1,218	999
Magnesium, mg	349	277	346	295	331	283
Iron, mg	13.6	10.7	14.5	13.0	12.3	9.6
Zinc, mg	11.4	8.3	10.1^2^	7.2^2^	10.2	8.0
Copper, mg	1.5	1.1	1.8^2^	1.5^2^	2.1	1.7
Selenium, μg	65	47	-	-	31	20
Iodine, µg	134	105	105	94	30	25

1 From National Survey, all adults’ mean intakes 17–64 years (*n* = 1,377).

2 From National Survey, all participants’ mean intakes 7–64 years (*n* for men 1,001 and *n* for women 948).

Note: In Estonia, sodium intake does not include salt/sodium from recipes.

Color coding of cells: Green – above RI; yellow – below RI; red – high intake compared to Nordic nutrition recommendations (1).

In general, mean reported intakes of most vitamins and minerals were above R1 in the Nordic countries (Table 4), but not to the same extent in the Baltic countries (Table 5). Mean vitamin D and folate intakes were low (marked as yellow cells) among most population groups, while mean intake of sodium was high (marked as red cells). Mean iron intake was lower than RI among women in all countries. Mean potassium intake was low in Sweden and Iceland, as well as in the Baltic countries. Further information on the intakes of nutrients in the Nordic and Baltic countries can be found in the Supplementary material. In the Supplementary material, nutrient intakes are divided by age groups and include information on children. The Supplementary material was put together as a background document for the authors of the different nutrient chapters in the NNR 2022 project.

The mean daily consumption of the different food groups in Nordic and Baltic countries is presented in Tables 6 and 7, respectively. The intakes differ both between countries and within countries, with large SDs. In addition, the definition of the food groups differed between the countries, making comparisons difficult. The mean intake of fruit and berries ranged between 100 and 235 g per day with the highest intakes in Estonian women (235 g), and the lowest intakes among men in Iceland and Sweden (around 100 g). The mean intake of vegetables was in general a bit higher and ranged between 150 and 200 g per day. The highest consumption of vegetables was reported for Danish and Latvian men (around 200 g). The mean milk and dairy product consumption ranged between 120 and 480 g per day, with the lowest intake among women in Lithuania (124 g) and the highest intake in Finnish men (480 g). However, the intake in Finland included cheese. The daily mean intake of potatoes ranged between 50 (Norwegian women) and 130 g (Swedish men). The mean intake of fish and seafood was highest in Norway, men consumed around 80 g per day and women around 60 g per day. The intake in Iceland was 55 g per day for men and 40 g per day for women. In the rest of the Nordic countries, the consumption ranged between 30 and 40 g per day, while in the Baltic countries, it was between 25 and 35 g per day. The intake of both pulses and nuts and seeds was very low in the Nordic and Baltic countries. The mean daily intake of meat and meat products varied roughly between 100 and 200 g per day, with higher reported intakes in men than in women. The highest consumption was reported for Latvian men (224 g per day).

**Table 6:** Consumption of selected food groups, g/day (mean and SD or 95% Confidence Interval (Finland)) in the Nordic countries. Data is retrieved from respective country’s national dietary survey in adults. Intake weight as consumed, unless otherwise described in footnotes

	Denmark 2011 (18–75 y)^1^		Finland 2017 (18–74 y)^2^		Iceland 2010 (18–80 y)		Norway 2010 (18–70 y)^3^		Sweden 2010 (18–80 y)^4^	
	Men (n = 1464)	Women (n = 1552)	Men (n = 780)	Women (n = 875)	Men (n = 632)	Women (n = 680)	Men (n = 862)	Women (n = 925)	Men (n = 792)	Women (n = 1005)
**Beverages**	2242 (886)	2129 (768)	1750	1774						
Soft drinks, cordials etc	258 (755)	181 (566)	117 (89–145)^5^ 38 (28–48)^6^	62 (52–72)^21^ 24 (17–30)^22^	274 (358)	205 (317)	282 (396)	202 (329)	132 (234)	95 (157)
Juice	59 (101)	54 (83)	51	43	89 (159)	90 (141)	114 (178)	100 (149)	84 (100)	52 (88)
Coffee	706 (553)	550 (467)	493 (466–521)	382 (357–406)	404 (432)	272 (279)	591 (508)	454 (452)	370 (290)	311 (256)
Tea	91 (220)	184 (335)	81 (68–94)	145 (122–167)	40 (114)	72 (151)	108 (251)	238 (355)	88 (161)	145 (218)
**Cereals**	249 (90)	189 (65)	149 (142–157)	111 (107–115)			272	179	219 (117)	165 (92)
Bread	157 (75)	121 (53)	107	74	105 (70)	85 (51)	227 (121)	144 (80)	102 (55)	75 (41)
Other grains							45 (62)	35 (41)		
Breakfast cereals	22 (30)	13 (19)	5	4	16 (26)	12 (18)			14 (22)	10 (16)
Porridge	11 (43)	14 (42)	92	81	32 (81)	26 (58)			42 (85)	34 (63)
**Potatoes**	118 (99)	66 (53)	85	62	72		83 (80)	50 (57)	133 (115)	73 (66)
**Vegetables**	191 (122)	206 (115)	177 (168–186)	191 (181–201)	122 (104)	117 (96)	154 (106)	155 (105)	169 (104)	182 (98)
Pulses (legumes)	1 (5)	1 (6)	12 (9–15)	14 (11–17)					12 (25)	12 (22)
**Fruits, and berries**	166 (149)	212 (143)	135 (123–146)	189 (175–203)	101 (118)	136 (120)	168 (155)	189 (143)	105 (112)	147 (108)
**Nuts and seeds**	3 (10)	5 (11)	7 (6–8)	9 (7–10)					4 (13)	5 (12)
**Fish and seafood**	40 (49)	34 (31)	36 (30–42)	27 (25–30)	55 (72)	38 (48)	79 (101)	56 (71)	43 (47)	37 (36)
**Meat and meat products**	172 (88)	99 (53)			167 (122)	96 (66)	181 (126)	116 (78)		
Read meat	172 (88)	99 (53)	138 (130–147)	71 (67–75)	33 (54)	23 (39)	149	92	112 (66)	68 (41)
Poultry	29 (37)	24 (27)	43 (37–49)	36 (31–41)			32	24	23 (32)	20 (25)
**Milk and dairy products**	337 (274)	273 (197)	480 (449–512)	394 (373–415)	353 (266)	251 (182)	384 (354)	249 (237)	267 (231)	227 (171)
**Cheese**	47 (35)	41 (31)					46 (44)	42 (38)	25(28)	25 (28)
**Eggs**	26 (22)	23 (17)	24 (22–27)	24 (21–27)	14 (27)	10 (18)	28 (42)	23 (33)	14 (23)	14 (20)
**Fats and oils**	47 (25)	35 (19)	53 (50–55)	38 (37–40)			39 (28)	24 (19)		
Spreads	18 (19)	11 (13)	18	13	14 (16)	10 (12)			13 (13)	10 (9)
Margarine			14	8,5	4 (5)	3 (4)				
Vegetable oil			10	8,6	2 (4)	2 (6)				
**Sweets and sweet bakery products**										
Cakes and biscuits	48 (51)	42 (41)	32 (30–34)	33(30–35)	52 (70)	43 (54)	36 (61)	34 (48)	33 (49)	39 (33)
Sweets	38 (35)	35 (28)			17 (30)	17 (25)			10 (19)	13 (22)

1 The food group Cereals contains both food ingredients and prepared foods such as bread. The food group Meat and meat products includes only red meat (not incl. poultry). Meat and fish are reported in raw weight, except sausages, cold cuts, and canned/smoked fish products. Potatoes – amount per se as well as part of dishes. Pulses – dry weight. The group Spreads includes only spreads on bread. Eggs also include eggs from the dishes.

2 All food groups except drinks include the contribution from dishes. Meat and fish are reported in raw weight, except sausages and cold cuts.

3 Meat and fish are reported in raw weight.

4 The following food groups include the contribution from dishes, Potatoes, Vegetables, Fruits, and berries, Fish and shellfish, meat, and poultry. The group Spreads includes only spreads on bread.

5 Sugar-sweetened beverages.

6 Artificially sweetened beverages.

**Table 7:** Consumption of selected food groups, g/day (mean and SD) in the Baltic countries. Data are retrieved from respective country’s national dietary survey in adults

	Estonia 2014 (18–74 years)^1^		Latvia 2020 (19–64 years)^2^		Lithuania 2019 (19–75 years)	
	Men (*n* = 907)	Women (*n* = 1,806)	Men (*n* = 470)	Women (*n* = 541)	Men (*n* = 1,348)	Women (*n* = 1,562)
**Beverages**			649 (478)	534 (339)		
Soft drinks, cordials, etc.	82 (159)	40 (137)	128	61	74 (198)^3^	36 (102)^3^
Juice	57 (115)	35 (117)	51	38		
Coffee	261 (199)	252 (252)	303	298	348 (258)^4^	356 (238)^4^
Tea	212 (218)	240 (322)	210	179		
**Cereals (grains)**			256 (139)	189 (109)	255 (181)	196 (142)
Bread	92 (51)	61 (52)	101	72		
Other grains	56 (61)	38 (64)	48	32		
Breakfast cereals	4 (13)	3 (16)	4.1	2.3		
Porridge	71 (93)	83 (138)	1.8	1.3		
**Potatoes**	126 (88)	78 (88)	125 (107)	85 (87)		
**Vegetables**	150 (88)	142 (127)	205 (163)	194 (138)	189 (144)	182 (142)
Pulses (legumes)	7 (14)	5 (22)	18	17		
**Fruits and berries**	182 (184)	235 (260)	127	165	150 (182)	170 (172)
	202 (195)^5^	257 (275)^5^				
**Nuts and seeds**	3.6 (12)	4.2 (17)	5.5	7.5		
**Fish and seafood**	29 (41)	23 (44)	36 (72)	26 (48)	29 (71)	27 (59)
**Meat and meat products**			224 (133)	136 (84)	174 (136)	111 (100)
Red meat	61 (44)	36 (47)	63	37		
Poultry	24 (40)	17 (39)	50	36		
Meat products (both red meat and poultry)	23 (32)	14 (31)	111	63		
**Milk and dairy products**	294 (220)	252 (223)	224 (222)	185 (156)	140 (163)	124 (131)
**Cheese**	20 (22)	15 (23)				
**Eggs**	26 (31)	20 (31)	40 (49)	31 (36)	34 (64)	23 (48)
**Fats and oils**			13 (13)	11 (11)	12 (18)	9 (14)
Butter	7.1 (9.4)	5.8 (10)	6.5	5.6		
Oil	8.8 (6.7)	6.1 (7)	1.4	1.6		
Other fats (margarine and mayonnaise)	10 (12)	7 (12)	4.4	3.4		
**Sweets and sweet bakery products**						
Cakes and biscuts	46 (59)	43 (71)				
Sweets	44 (38)	41 (50)	32	26	24 (40)	20 (36)

1 The food group soft drinks, cordials, etc. also includes energy drinks, near water, nectars, and fruit drinks. The food group sweets includes sugar and sweet snacks (including honey, candy, chocolate, jam, milk, desserts, ice-cream, etc.).

2 Data from the study of salt and iodine consumption in the adult population in Latvia, 2020. The food group beverages does not include juices. The food group cereals (grains) also includes bakery products, like cakes, cookies, etc. The food group vegetables also includes pulses (legumes) and nuts. The food group other fats (margarine and mayonnaise) does not include mayonnaise. The food group sweets includes sugar, sweet, and snacks (including honey, candy, chocolate, jam, milk, desserts, ice-cream, etc.).

3 Fruit and vegetable juices, sugar sweetened and artificially sweetened drinks, and bottled water.

4 Coffee, tea, and cocoa.

5 Including fruit-berry preserves and dried.

## Discussion

The Nordic and Baltic countries share not only similarities but also differences in food culture, which is reflected in differences in average food consumption and nutrient intakes between the countries. Difference in intake data may also be related to the dietary assessment method and number of surveyed days, misreporting in the dietary survey, participation rates, and the food composition databases used in different surveys. The food grouping differed between the countries, which makes comparability challenging. For example, in Finland, the dairy product group included all dairy products, including cheese, consumed individually or as part of a dish. The Swedish data, however, only included milk and milk products consumed individually, and cheese was reported as a separate food group. Furthermore, in Denmark, Finland, and Norway, the intake of fish and meat is reported partly as raw weight and not as consumed as in the other countries. For the food groups, the intake range was large in all countries with large SDs, implying large interindividual differences in food consumption within each country. The fact that the surveys in different countries were conducted over a long time period (2007–2020) may have influenced the observed differences in intakes because of the changing food markets.

Nutrient intakes are easier to compare between the countries since the units of nutrients are harmonized between the countries. Differences in reported nutrient intakes between the countries are due to real differences in nutrient contents of foods, fortification policies, as well as food culture differences, which is reflected in differences in intake data relating to food preferences and culinary traditions. Finland reported the highest mean intake of selenium, and this is a real difference since selenium-fortified fertilizers are used for crops, which has increased both selenium intakes and selenium status in the Finnish population (19). The database values and calculation procedure of the nutrient intakes in the surveys also influence intakes and contribute to differences between countries. For example, tiamin and folate values for boiled potatoes are lower in Estonian database (0.036 mg and 12 µg per 100 g, respectively) (20) compared with the Swedish database (0.06 mg and 18 µg per 100 g, respectively) (21).

Reported daily mean intakes of most vitamins and minerals were above RI in the Nordic countries, but not to the same extent in the Baltic countries. The lower intakes in the Baltic countries may partly be due to not only missing or lower nutrient values, as illustrated by the tiamin and folate values for boiled potatoes, in the food composition databases, but also underreporting since the reported intake of energy was low. Reported intakes of vitamin D and folate were low in most population groups, while intake of sodium was too high. Estonia reported the lowest intake of sodium, which may be related to the fact that sodium from the salt normally used in standard recipes is lacking in the Estonian food database. Finland reported the highest intake of vitamin D, while Denmark reported the lowest intake. The high intake in Finland is consistent with a successful voluntary vitamin D fortification policy, while few products are fortified with vitamin D in Denmark (22). Iron intake was below RI among women in all countries; however, this may not necessarily mean that the intake is deficient in a large proportion of the population since the iron needs are unevenly distributed (1). As an example, in the national dietary survey of Sweden (8), 29% of the women of childbearing age between 18 and 44 years had low iron stores (serum ferritin < 15 µg/L) (23), while the mean iron intake in the same age groups was around 9 mg, well below the RI (15 mg) for iron. In fact, the 95th percentile intake was around 15 mg (5). Potassium intake was low in Sweden and Iceland, as well as in the Baltic countries. A mean micronutrient intake above RI indicates a probable low risk for inadequacy but does not exclude that individuals have inadequate intakes. To make a thorough evaluation of the micronutrient inadequacy, the proportion of the population with an intake below AR has to be calculated (1), and this is beyond the scope of this article.

The percentage contribution of macronutrients, including alcohol intake, to total energy was roughly similar among the populations in the Nordic countries as well as in Estonia and in accordance with the RI range in the NNR 2012 (1). In Latvia and Lithuania, the contribution from alcohol was not included in the presented figures, making comparison with other countries difficult. However, the reported intake of energy from fat was numerically higher and lower from carbohydrates, compared to the rest. The reported intake of SFA was higher than the recommendation (maximum 10 E%) in all countries, and this is consistent with the situation in most other European countries (24). Fiber intake was lower than the recommendation in all countries.

Recently, food calculations of a standardized 7 day menu rendering nutrient intakes and amount of food groups were performed by the dietary survey teams in the Nordic countries (25). Although the same input items were used for the calculations by all the countries, there were many differences in the results, both for nutrients and food groups. It was concluded that the differences were due to differences in food grouping, recipes in the food composition databases, fortification strategies in each country, calculation systems with different yield and retention factors, real differences in nutrient contents, and missing values in food composition databases (25). These are the same factors, apart from differences in food consumption patterns, causing the differences in reported intakes in the national dietary surveys compared here. This highlights the challenge to compare intake data between countries and the need to translate the DRVs and FBDG in the NNR into national FBDG to fit each country’s food culture as well as other aspects, such as environmental sustainability and toxicological aspects. The need and challenges of harmonized dietary data on a global level have previously been discussed (26). To facilitate future comparisons between the Nordic and Baltic countries, it would be of interest to harmonize food groupings, and the age groups reported on and ideally harmonization of survey methods would be preferred but maybe not feasible. In the present review, the intake of, for example, wholegrains, added sugar, and free sugar was not reported, since this information was missing in several of the countries. In a future comparison, it is advisable that wholegrains, added sugar, and free sugar are included. It would also be good if the countries would report nutrient intakes in a harmonized manner, including both mean intakes, SDs, and percentiles to be able to compare with DRVs.

## Conclusion

The nutrient intake and, especially, food consumption differ between the Nordic and Baltic countries because of differences in food consumption pattern and factors related to the dietary surveying, food grouping, food databases, and calculation procedures in each country. The broad intake data presented in this article will be used as a background document for the formulation of DRVs and FBDG within the NNR 2022 project.

## Supplementary Material

The Nordic Nutrition Recommendations 2022 – food consumption and nutrient intake in the adult population of the Nordic and Baltic countries
